# N-Methyl-D-Aspartate Receptors Involvement in the Gentamicin-Induced Hearing Loss and Pathological Changes of Ribbon Synapse in the Mouse Cochlear Inner Hair Cells

**DOI:** 10.1155/2018/3989201

**Published:** 2018-07-15

**Authors:** Juan Hong, Yan Chen, Yanping Zhang, Jieying Li, Liujie Ren, Lin Yang, Lusen Shi, Ao Li, Tianyu Zhang, Huawei Li, Peidong Dai

**Affiliations:** ^1^ENT Institute and Otorhinolaryngology Department of Affiliated Eye and ENT Hospital, State Key Laboratory of Medical Neurobiology, Fudan University, Shanghai 200031, China; ^2^Department of Otorhinolaryngology-Head and Neck Surgery, Huashan Hospital, Fudan University, Shanghai 200040, China; ^3^NHC Key Laboratory of Hearing Medicine, Fudan University, Shanghai 200031, China; ^4^Department of Otolaryngology Head and Neck Surgery, Affiliated Drum Tower Hospital of Nanjing University Medical School, Jiangsu Provincial Key Medical Discipline (Laboratory), Nanjing 210008, China; ^5^Institutes of Biomedical Sciences, The Institutes of Brain Science and the Collaborative Innovation Center for Brain Science, Fudan University, Shanghai 200032, China; ^6^Shanghai Engineering Research Centre of Cochlear Implant, Shanghai 200031, China

## Abstract

Cochlear inner hair cell (IHC) ribbon synapses play an important role in sound encoding and neurotransmitter release. Previous reports show that both noise and aminoglycoside exposures lead to reduced numbers and morphologic changes of synaptic ribbons. In this work, we determined the distribution of N-methyl-D-aspartate receptors (NMDARs) and their role in the gentamicin-induced pathological changes of cochlear IHC ribbon synaptic elements. In normal mature mouse cochleae, the majority of NMDARs were distributed on the modiolar side of IHCs and close to the IHC nuclei region, while most of synaptic ribbons and *α*-amino-3-hydroxy-5-methyl-4-isoxazolepropionic acid receptor (AMPAR) were located on neural terminals closer to the IHC basal poles. After gentamicin exposure, the NMDARs increased and moved towards the IHC basal poles. At the same time, synaptic ribbons and AMPARs moved toward the IHC bundle poles on the afferent dendrites. The number of ribbon synapse decreased, and this was accompanied by increased auditory brainstem response thresholds and reduced wave I amplitudes. NMDAR antagonist MK801 treatment reduced the gentamicin-induced hearing loss and the pathological changes of IHC ribbon synapse, suggesting that NMDARs were involved in gentamicin-induced ototoxicity by regulating the number and distribution of IHC ribbon synapses.

## 1. Introduction

Cochlear inner hair cell (IHC) ribbon synapses play an important role in sound encoding and glutamate release. The IHC ribbon synapses are the first afferent synaptic connection in the hearing pathway, and they are located between the IHCs and the terminals of spiral ganglion neurons (SGNs). Bursts of synaptic activity are induced through periodic excitation of IHCs by mechanisms that are intrinsic to the cochlea [1], resulting in IHC Ca^2+^ spikes, glutamate release, and ultimately bursts of action potentials in SGNs that are carried to the brain by auditory nerve fibers. Cochlear synaptic ribbon pairs consist of presynaptic ribbons, to which many synaptic vesicles are connected, and postsynaptic *α*-amino-3-hydroxy-5-methyl-4-isoxazolepropionic acid receptors (AMPARs), which are glutamate receptors that mediate the excitatory postsynaptic currents of the SGNs' afferent dendrites [2].

Cochlear ribbon synapses are very sensitive to aminoglycoside and noise-induced injury [3–6]. Liberman et al. reported the reorganization of synaptic ribbon locations, the loss of synaptic ribbons, and the downregulation of AMPAR expression in the peripheral terminals after noise exposure [7]. Liu et al. found that moderate ototoxicity in mice leads to reduced numbers and morphologic changes in the synaptic ribbons, which are accompanied by mild hearing loss but no significant loss of HCs or SGNs in the cochlea [8]. However, the mechanism behind these pathological changes in cochlear IHC ribbon synapse remains unclear.

In the mammalian inner ear, almost all SGNs express another glutamate receptor, N-methyl-D-aspartate receptor (NMDAR), in addition to AMPAR [9–12]. In the developing cochlea, NMDARs in the IHC-SGN synapses enhance the spontaneous activity and promote the survival of SGNs [13]. NMDARs have also been shown to be involved in regulating the number of surface AMPARs on the cell membrane of auditory neurons in cultured neurons and to be involved in the response to acoustic stimulation [14]. However, the function of NMDARs was not clear in the IHC-SGN synapses of the mature cochlea.

Several studies suggest that NMDARs play a role in ototoxicity in the cochlea [15–17]. Aminoglycosides generate excess free radicals in the cochlea that damage both sensory HCs and SGNs, resulting in permanent hearing loss [18]. By using a severe hearing loss model with a large dose of aminoglycoside, in which both HCs and SGNs were severely injured, Basile et al. demonstrated that the NMDA antagonist dizocilpine maleate (MK801) could attenuate aminoglycoside-induced damage to SGNs and IHCs [15]. However, in this severe hearing loss model, the detailed pathological changes in the afferent synaptic connection between the IHCs and SGNs could not be evaluated due to the extensive injury to the HCs and SGNs. In this study, we used a low dose of gentamicin (100 mg/kg bodyweight) [3, 8, 19] to obtain a mild hearing loss model in which most of the HCs survived. We systematically analyzed the detailed morphological configuration, distribution, and number of presynaptic ribbons, postsynaptic AMPARs, and NMDARs in the cochlear IHC-SGN synapses of adult mice. To determine the role of NMDARs in the IHC-SGN synaptic plasticity in response to ototoxicity, we used the NMDAR antagonist MK801, which prevented gentamicin-induced injury to the IHC ribbon synapse and thus prevented hearing loss *in vivo*.

## 2. Materials and Methods

### 2.1. Animals

In total, 30 female C57BL/6J mice with documented dates of birth (5 weeks old) were obtained from the Chinese Academy of Medical Sciences Animal Center (Shanghai, China). All mice were housed with free access to food and water at the Experimental Animal Center, Shanghai Medical College of Fudan University, China. No outer or middle ear pathologies were observed. The animals were divided randomly into three groups. The first group was injected daily with a low dose of gentamicin (100 mg/kg, Sigma-Aldrich, USA) in saline intraperitoneally (i.p.) for 4 or 7 consecutive days [3, 8, 19, 20]. The second group of animals received daily i.p. injections of gentamicin (100 mg/kg in saline) and the NMDAR antagonist MK801 (0.2 mg/kg in saline, Sigma-Aldrich, USA) for 4 or 7 consecutive days [17, 21, 22]. The mice in the third group served as the control group and received daily injections of equivalent volumes of normal saline. This study was carried out in strict accordance with the “Guiding Directive for Humane Treatment of Laboratory Animals” issued by the Chinese National Ministry of Science and Technology in September 2006. We performed all animal procedures according to protocols that were approved by the Shanghai Medical Experimental Animal Administrative Committee and were consistent with the National Institute of Health's Guide for the Care and Use of Laboratory Animals. All efforts were made to minimize suffering and reduce the number of animals used [23].

### 2.2. Assessment of Auditory Function

Auditory brainstem response (ABR) tests were performed in a sound-attenuating chamber to determine auditory thresholds. Animals were anesthetized via i.p. injections with ketamine (100 mg/kg) and xylazine (25 mg/kg). Specific auditory stimuli (clicks and 4, 8, 16, and 24 kHz tone bursts) were measured using a Tucker-Davis Technology System 3 (Tucker-Davis Technologies, Gainesville, FL, USA) as described previously [23, 24]. ABR stimuli consisted of 5 ms tone pips with a 0.5 ms rise-fall time delivered at 30 stimuli. The ABR threshold was defined as the lowest stimulus level at which a repeatable morphology could be identified in the response waveform (at least two consistent peaks). The ABR wave I peak-to-peak amplitude was computed by off-line analysis of stored waveforms as previous studies [12, 25]. All ABR tests were performed on mice on the following day after the injections with gentamicin alone or in combination with MK-801 (i.e., ABR was performed on day 5 or day 8).

### 2.3. Cochlear Tissue Processing and Immunostaining

After the ABR recordings, the mice were sacrificed by cervical dislocation and then decapitated. The temporal bone was removed, and the cochlea was quickly separated. The round and oval windows were opened and perfused with 4% paraformaldehyde in phosphate-buffered saline (PBS) at pH 7.4 and postfixed in the same solution for 2 h at room temperature. All cochleae were decalcified in 10% ethylene diamine tetraacetic acid (EDTA) solution. As quickly as possible, the cochlear shell and spiral ligament were removed under a dissecting microscope in 0.01 mM PBS solution. The vestibular membrane and tectorial membrane were removed from the basal membrane. The apical, middle, and basal turns from the cochlear basilar membrane were processed for immunofluorescent staining with antibodies against myosin 7a. Considering the fact that ribbon synapses near the basal turn of the cochlea are most susceptible to ototoxicity exposure [3, 8, 19], in this paper, only the synaptic elements of the cochlear middle-basal turn (51%–75% of the cochlear length from the apex) were quantified to determine the gentamicin-induced synaptic pathology.

Specimens were blocked with 10% donkey serum in 10 mM PBS with 0.3% Triton X-100 for 1 h at room temperature and then incubated with primary antibody for 48 h at 4°C. The primary antibodies included mouse anti-C-terminal-binding protein-2 (CtBP2) (612044; BD Biosciences, USA) at 1 : 500 dilution; mouse anti-glutamate receptor 2, extracellular, clone 6C4 (GluA2) (MAB397; Millipore, Germany) at 1 : 2000 dilution; rabbit anti-NMDAR1 (GluN1) (AB9864R; Millipore, Germany) at 1 : 1000 dilution; chicken anti-200 kD neurofilament heavy chain (NF) (cat. number ab72996; Abcam, UK) at 1 : 1500 dilution; and rabbit anti-myosin 7a polyclonal (Proteus BioSciences, USA) at 1 : 500 dilution. On the following day, the appropriate Alexa Fluor-conjugated secondary antibodies were incubated overnight at 4°C. Nuclear staining was performed with DAPI (1 : 800 dilution; Sigma-Aldrich, USA). Negative controls were performed by omitting the primary antibodies [26–28].

### 2.4. Confocal Microscopy Imaging

For confocal microscopy imaging, a laser scanning confocal microscope was used (SP5; Leica Microsystems, Biberach, Germany) with a 63x oil immersion objective lens. Excitation wavelengths were 488, 568, and 647 nm, and local images were magnified digitally by 2.74-fold. In each region, 10 to 12 IHCs were typically assessed. Sequence scanning was performed at an interval of 0.50 *μ*m [27]. The laser excitation power and microscope emission and detection settings were maintained across different observations. The resulting confocal image series (*z*-stack) contained a three-dimensional (3D) record of the imaged information in the entire volume of the explant. The 3D-reconstructed *z*-stack was viewed, rotated, and “sliced” to provide final images and movies as necessary using the software from a TCS SP8 confocal laser-scanning microscope (Leica, Heidelberg, Germany).

### 2.5. Synapse Counts and Positioning Analysis

**Figure 1:**
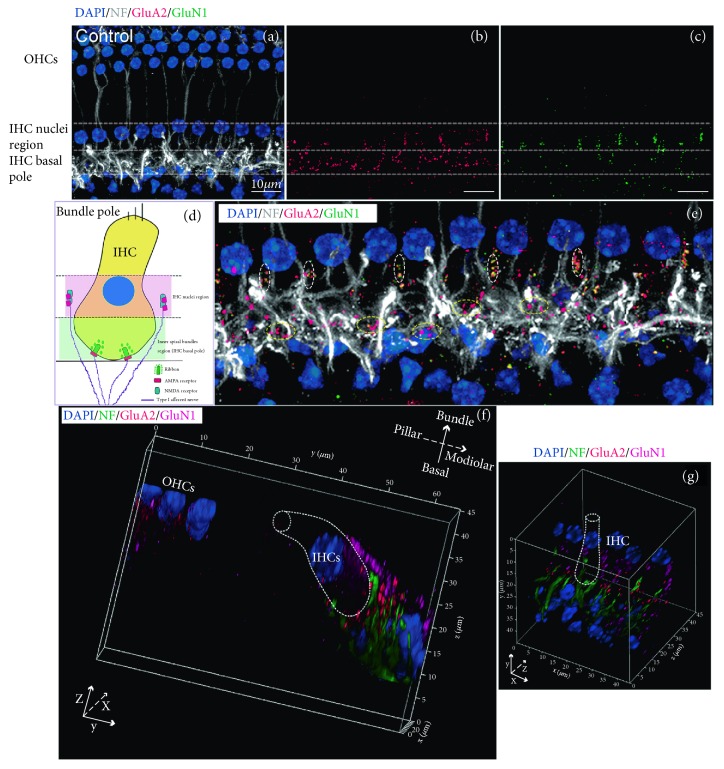
The different distributions of NMDARs and AMPARs at afferent dendritic terminals around IHCs in the normal mature cochlea. Postsynaptic AMPARs, NMDARs, and nerve fibers were identified by immunostaining for GluA2 (red), GluN1 (green), and neurofilament (NF) (grey), respectively. Nuclei were labeled with DAPI (blue). (a) The ISBs appeared as a dense meshwork of neuronal processes at the basal pole region of the IHCs. (b) AMPAR patches were mostly distributed among the ISBs around the basal poles of the IHCs. (c) Most of the NMDAR patches were distributed on the neural dendritic terminals, which were near the IHC nuclei region. (d) A diagram of the different locations of NMDARs and AMPARs at type I ANFs under physiological conditions. To count the number of synaptic elements, we drew one line along the top of the IHC nuclei and another line along the bottom of the ISBs. Then the field between these two lines was divided into two parts, the “IHC nuclei region” and the “IHC basal pole region.” The IHC nuclei region was defined as the half region proximal to the nuclei and the bundle poles of the IHCs, and the IHC basal pole region was defined as the half region proximal to the ISBs and the basal poles of the IHCs. (e) Enlarged view of merged images (a), (b), and (c). Most of the NMDAR patches were distributed on the neural dendritic terminals between the adjoining IHCs and closer to the IHC nuclei region, while most of the AMPAR patches were observed among the ISBs around the basal poles of the IHCs. In the IHC nuclei region, some NMDARs colocalized with GluA2-positive AMPAR puncta (the dashed white circles), which were smaller than AMPAR patches in the IHC basal pole region (the dashed yellow circles). (f, g) The 3D-reconstructed images showing spatial distributions of AMPARs and NMDARs in SGN fibers around the IHCs. Postsynaptic AMPARs, NMDARs, and nerve fibers were identified by immunostaining for GluA2 (red), GluN1 (magenta), and NF (green), respectively. Nuclei were labeled with DAPI (blue). IHC: inner hair cell; OHC: outer hair cell; ISBs: inner spiral bundles; ANFs: afferent nerve fibers. Scale bar = 10 *μ*m.

The contrast and brightness of the images were processed using Adobe Photoshop CS6. Synapse counts from the confocal images were performed using ImageJ (NIH, USA). The ribbon pairs were identified by the positive colocalization for double staining with CtBP2 and GluA2. Immunofluorescently identified AMPAR patches and NMDAR patches were counted at afferent nerve fibers (ANFs). All IHCs observed in 2 or 3 image fields were counted [26, 27]. The total numbers of puncta and patches were divided by the total numbers of IHC nuclei to obtain the average number of ribbons and glutamate receptors for each IHC. To describe the distribution of ribbons in IHCs and glutamate receptors (AMPARs and NMDARs) in afferent dendritic terminals, the concepts of basal and bundle poles of IHCs were introduced [29]. To count the number of synaptic elements, we drew one line along the top of the IHC nuclei and another line along the bottom of the inner spiral bundles (ISBs). Then the field between these two lines was divided into two parts, the “IHC nuclei region” and “IHC basal pole region.” As shown in Figure 1(d), the IHC nuclei region was defined as the half region proximal to the nuclei and the bundle poles of the IHCs, and the IHC basal pole region was defined as the half region proximal to the ISBs and the basal poles of IHCs. The synaptic elements located between the IHC nuclei region and IHC bundle pole were also counted within the IHC nuclei region for convenience.

### 2.6. Statistical Analysis

All data are shown as means ± SE. Statistical analyses were conducted using Microsoft Excel and GraphPad Prism® 6 software. In all experiments, *n* represents the number of replicates. Two-tailed, unpaired Student's *t*-tests were used to determine statistical significance when comparing two groups, and one-way ANOVA followed by Dunnett's multiple comparisons test was used when comparing more than two groups. A value of *p* < 0.05 was considered to indicate statistical significance.

## 3. Results

### 3.1. The Distributions of Ribbons, NMDARs, and AMPARs at the IHC-SGN Synaptic Connection in the Mature Cochlea under Physiological Conditions

**Figure 2:**
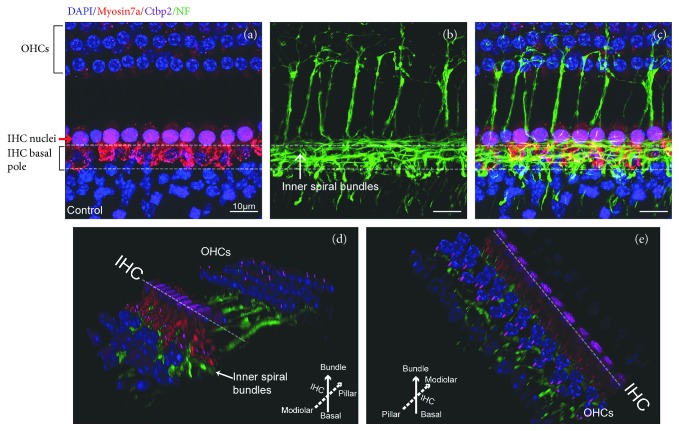
Spatial distribution of cochlear ribbons in IHCs and their relationship with inner spiral bundles in the normal mouse mature cochlea. The HCs, presynaptic ribbons, and nerve fibers were identified by immunostaining for myosin 7a (red), Ctbp2 (magenta), and neurofilament (NF) (green), respectively. Nuclei were labeled with DAPI (blue). (a) Ctbp2-positive ribbons (magenta puncta) were distributed at the basal poles of the IHCs and below the IHC nuclei. The IHC nuclei are marked with a red arrow. (b) NF staining showed that the ISBs appeared as nets of nerve fibers that include radially directed dendritic terminals of type I ANFs and efferent fibers of the olivocochlear system. (c) Merged images of (a) and (b). The IHC basal poles were surrounded by ISBs. (d, e) The 3D-reconstructed images showing the spatial relation among IHCs, presynaptic ribbons, and ISBs. (d) View from the modiolar side to the pillar side. (e) View from the pillar side to the modiolar side. IHC: inner hair cell; OHC: outer hair cell; ISBs: inner spiral bundles; ANFs: afferent nerve fibers. Scar bar = 10 *μ*m.

We first evaluated the morphological features, distribution, and numbers of ribbons, NMDARs, and AMPARs in the IHC-SGN synaptic connection of the adult mouse cochlea. CtBP2 was used as a marker of presynaptic ribbons. GluN1 and GluA2 were used as markers of NMDARs and AMPARs, respectively. Consistent with a previous study [2], in normal mature cochleae, the CtBP2-positive IHC ribbons were distributed on the cell membrane at the basal poles of IHCs, close to the ISBs (Figures 2(a)–2(e)). The ISBs appeared as a dense meshwork of neuronal processes around the basal poles of the IHCs (Figures 2(b)–2(e)), and these mostly included radially directed dendritic terminals of afferent type I ANFs and the efferent fibers [27, 30].

We observed different distributions of NMDARs and AMPARs at the IHC-SGN synapses in normal mature cochleae. In order to quantify these synaptic elements, we defined the IHC nuclei region and IHC basal pole region (see Figures 1(a) and 1(d) and Materials and Methods). The majority of GluA2-positive AMPAR patches were observed in ISBs, which were found in the basal pole region of IHCs (Figures 1(a)–1(e)), while most of GluN1-positive NMDAR patches were distributed on the neural dendritic terminals that were closer to the IHC nuclei region (Figures 1(c) and 1(e)). In the IHC nuclei region, some NMDARs colocalized with GluA2-positive AMPAR puncta, which were smaller than the AMPAR patches in the IHC basal pole region. Counts of NMDARs in the mature mouse cochlear middle-basal turn yielded averages of 1.00 ± 0.22 and 5.40 ± 0.27 per IHC in the IHC basal pole region and the IHC nuclei region, respectively (Supplementary Table 2). The average numbers of AMPARs were 13.70 ± 0.42 and 1.20 ± 0.13 per IHC in the IHC basal pole region and the IHC nuclei region, respectively (Supplementary Table 1). The 3D-reconstructed images showed that in normal mature mouse cochleae, the majority of NMDARs were distributed on the modiolar side of IHCs and close to the IHC nuclei region, while the AMPARs were distributed at the IHC basal pole region, on the modiolar and pillar sides of IHCs (Figures 1(f) and 1(g) and Supplemental video 1).

### 3.2. A Low Dose of Gentamicin-Induced Moderate Hearing Loss and the Rearrangement of Cochlear IHC-SGN Synaptic Elements

**Figure 3:**
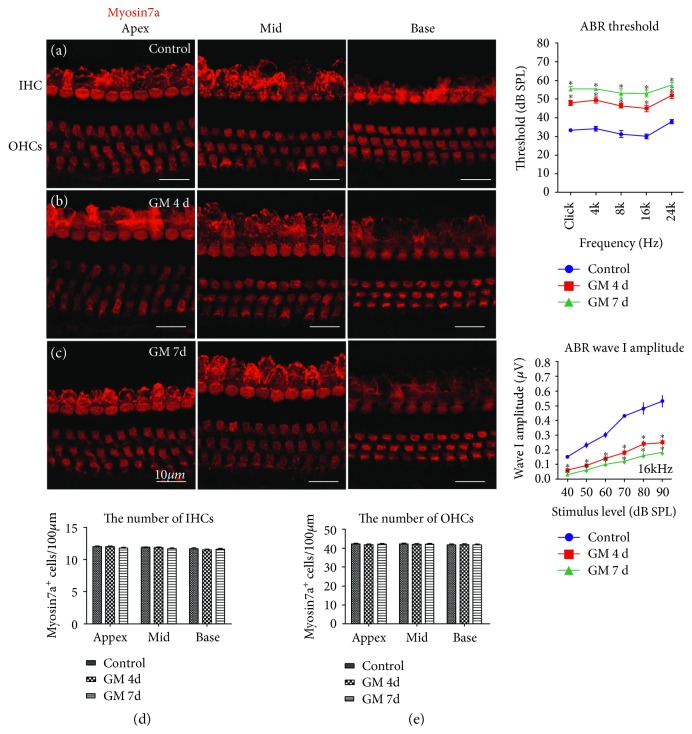
A low dose of gentamicin-induced moderate hearing loss with no obvious HC loss. The HCs were identified by immunostaining for myosin 7a (red) in the three turns of the cochlea. (a–c) After gentamicin treatment for 4 and 7 days, the morphology and arrangement of cochlear HCs were similar to the control group. (d-e) Quantitative data showed no HC loss in any of the three turns after gentamicin treatment. No significant difference was found in the number of IHCs or the three rows of OHCs among the control group and the gentamicin-treatment groups for 4 days or 7 days (*p* > 0.05). (f) A significant increase was observed in the ABR thresholds for clicks and for tone bursts at 4, 8, 16, and 24 kHz in the gentamicin-treated groups (^∗^ *p* < 0.05, versus the control group). (g) A significant decline was observed in the ABR wave I amplitudes at 16 kHz in the gentamicin-treated groups (^∗^ *p* < 0.05, *versus* the control group). GM 4 d, 7 d: gentamicin treatment for 4 days or 7 days; IHC: inner hair cell; OHC: outer hair cell. Scale bar = 10 *μ*m. ^∗^ *p* < 0.05.

In order to determine the mechanisms of synaptic pathology, the same dose of gentamicin (100 mg/kg) as used in previous studies was applied to the mice in this study [3, 8, 19]. Briefly, mice were injected daily with gentamicin (100 mg/kg) for 4 or 7 consecutive days, and mice receiving injections of normal saline served as controls. ABR tests were performed on the following day after gentamicin injections for 4 or 7 days. The mice were sacrificed, and the cochleae were isolated for immunostaining. Consistent with these previous reports [3, 8], we also found that a low dose of ototoxic gentamicin (100 mg/kg) led to moderate hearing loss and that this was associated with pathological changes in the presynaptic ribbons but with intact HCs and SGNs. Myosin 7a staining showed no obvious HC loss in any of the three turns after gentamicin treatment; however, moderate elevations in ABR thresholds for clicks and tone bursts at 4, 8, 16, and 24 kHz were found in the gentamicin-treated group (*p* < 0.05, Figures 3(a)–3(f)). The amplitude of ABR wave I, reflecting the synchronous summated neural activity of the auditory nerve and functional level of IHC synapses between IHCs and terminals of SGNs [25, 31], was significantly reduced in gentamicin-treated mice (Figure 3(g), *p* < 0.05).

**Figure 4:**
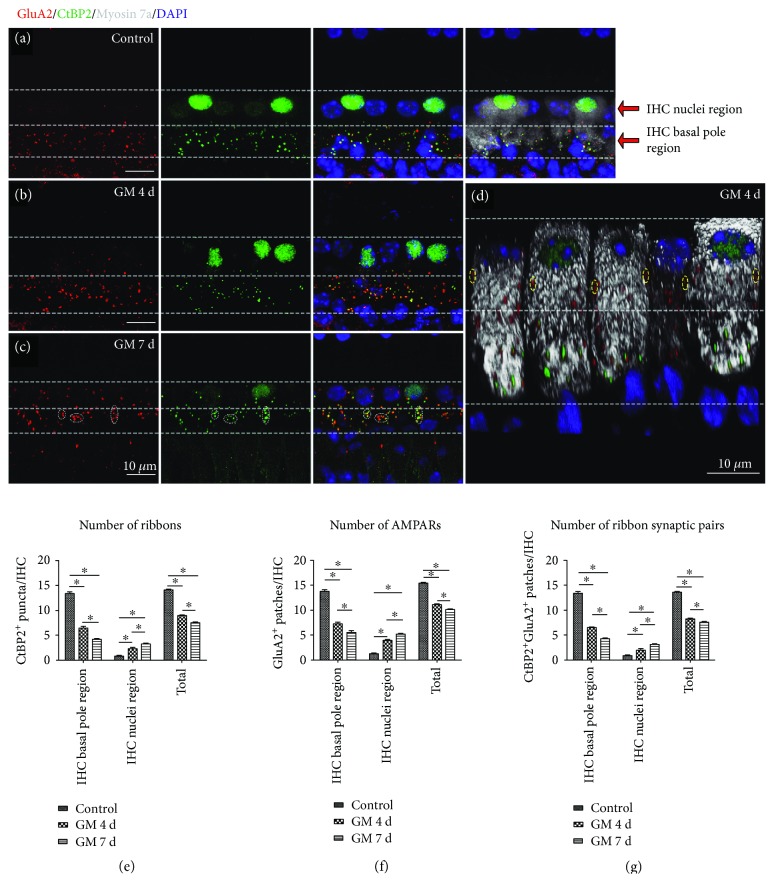
The number of cochlear ribbon synapses decreased and their locations changed from the basal pole region toward the nuclei region of the IHCs after gentamicin treatment. The IHCs were outlined by myosin 7a fluorescence in the cytoplasm (grey), and the nuclei were stained with DAPI (blue). The afferent synapses on the IHCs were labeled by immunostaining presynaptic ribbons with anti-CtBP2 (green) and the postsynaptic AMPARs with anti-GluA2 (red). (a) Ribbons and AMPARs were almost perfectly paired at the basal poles of the IHCs under physiological conditions. (b) After gentamicin treatment for 4 days, the ribbons and AMPARs moved towards the bundle poles of the IHCs. (c) After gentamicin treatment for 7 days, ribbons and AMPARs migrated towards the bundle poles of the IHCs, and some of them reached to or across the IHC nuclei region. The dashed white circles indicate some variegated ribbons and/or AMPARs at the IHC basal pole region. (d) The 3D-reconstructed image. The dashed yellow circles show orphan AMPARs near the bundle poles of the IHCs after 4 days of gentamicin treatment. (e) The numbers of CtBP2-positive ribbons. (f) The numbers of GluA2-positive AMPARs. (g) The numbers of CtBP2 and GluA2 double-positive ribbon synaptic pairs. GM 4 d, 7 d: gentamicin treatment for 4 days or 7 days; IHC: inner hair cell. Scale bar = 10 *μ*m. ^∗^ *p* < 0.05.

In normal mature cochleae, CtBP2-positive presynaptic ribbons were paired to GluA2-positive postsynaptic AMPARs, and the paired CtBP2/GluA2 double-positive patches were defined as “ribbon synaptic pairs.” In undamaged mouse cochleae, most of the ribbon synaptic pairs were distributed around the basal poles of the IHCs (Figure 4(a)). The average numbers of ribbon synaptic pairs were 13.30 ± 0.37 and 0.80 ± 0.10 per IHC in the IHC basal pole region and the IHC nuclei region, respectively, in the control group (Supplementary Table 1). In the gentamicin-treated group, the number of ribbon synaptic pairs was significantly decreased. Moreover, presynaptic ribbons and postsynaptic AMPARs were relocated toward the IHC bundle poles, some of which reached to or across the IHC nuclei region (Figures 4(b) and 4(c) and Supplemental Videos 3 and 4).

In the IHC basal pole region, gentamicin treatment induced a significant decrease in the number of ribbons (13.30 ± 0.37, 6.40 ± 0.37, and 4.20 ± 0.23 in the control, 4-day gentamicin, and 7-day gentamicin groups, resp.) (Figure 4(e) and Supplementary Table 1). The average number of AMPARs was also significantly decreased in the IHC basal pole region in gentamicin-treated cochleae compared with the control group (13.70 ± 0.42, 7.20 ± 0.39, and 5.50 ± 0.37 in the control, 4-day gentamicin, and 7-day gentamicin groups, resp.) (Figure 4(f) and Supplementary Table 1). These results indicate that there was a significant decrease in the number of presynaptic ribbons and postsynaptic AMPARs at the basal poles of the IHCs (Figures 4(e)–4(g)), and thus the number of ribbon synaptic pairs significantly decreased at the basal poles of the IHCs in the gentamicin-treated group compared with the control group (*p* < 0.05) (Figure 4(g)). We also observed some anomalously aggregated ribbons and/or AMPARs at the IHC basal pole region in the gentamicin-treated cochleae (Figure 4(c)), which is consistent with a previous report [8].

In the IHC nuclei region in normal cochleae, the average number of AMPARs, ribbons, and ribbon synaptic pairs were 1.20 ± 0.13, 0.80 ± 0.10, and 0.80 ± 0.10 per IHC, respectively. After gentamicin treatment, there was a significant increase in the number of relocated AMPARs, ribbons, and ribbon synaptic pairs in the IHC nuclei region (*p* < 0.05). After gentamicin treatment for 4 days, the numbers of AMPARs, ribbons, and ribbon synaptic pairs were 3.80 ± 0.28, 2.20 ± 0.32, and 1.90 ± 0.32 per IHC in the IHC nuclei region, respectively. After gentamicin treatment for 7 days, the numbers of AMPARs, ribbons, and ribbon synaptic pairs were 5.00 ± 0.31, 3.10 ± 0.28, and 3.00 ± 0.28 per IHC, respectively, in the IHC nuclei region (Figures 4(e)–4(g) and Supplementary Table 1). Moreover, many orphan AMPARs, which lack closely apposed presynaptic ribbons in the IHC, were observed in the IHC nuclei region after gentamicin treatment for 4 days (Figure 4(d)). More relocated ribbon synaptic pairs were found in the IHC nuclei region in the 7-day gentamicin group than the 4-day gentamicin group (Figure 4(g)), suggesting that the IHC ribbons matched up again with the earlier relocated postsynaptic AMPARs in the 7-day gentamicin group.

### 3.3. A Low Dose of Gentamicin-Induced Rearrangement of NMDARs at Nerve Terminals Innervating the IHCs

**Figure 5:**
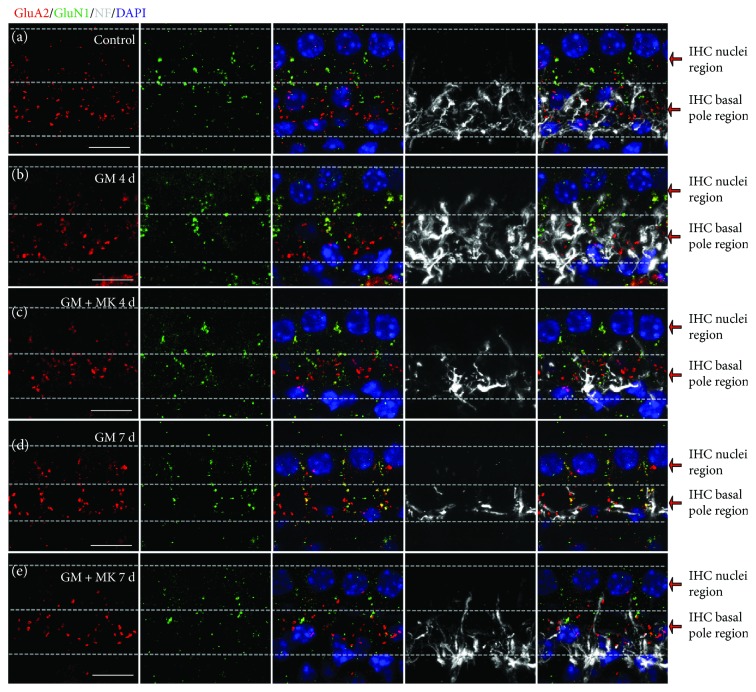
AMPARs and NMDARs were relocated on SGN dendrites after gentamicin exposure, and this was blocked by coinjection of the NMDAR antagonist MK801. Postsynaptic AMPARs, NMDARs, and nerve fibers were identified by immunostaining for GluA2 (red), GluN1 (green), and neurofilament (NF) (grey), respectively. Nuclei were labeled with DAPI (blue). (a) The different locations of NMDARs and AMPARs at afferent dendritic terminals in the normal mature cochlea. AMPAR patches were mostly observed on the ISBs around the basal poles of the IHCs, while NMDAR patches were almost all distributed on nerve terminals between the adjoining IHCs and closer to the IHC nuclei region. (b) After gentamicin treatment for 4 days, the AMPARs migrated upwards towards the bundle poles of the IHCs, while NMDARs migrated downwards towards the basal poles of the IHCs. (c) After the combined treatment with MK801 and gentamicin for 4 days, AMPARs and NMDARs were prevented from delocalizing to the dendritic terminals around the IHCs. (d) After gentamicin treatment for 7 days, the AMPARs and NMDARs had essentially switched locations along the dendritic terminals. Many colocalized AMPARs and NMDARs were observed laterally between the adjoining IHCs. (e) After the combined treatment with gentamicin and MK801 for 7 days, the gentamicin-induced rearrangements of AMPARs and NMDARs at the afferent dendritic terminals were partly blocked. GM 4 d, 7 d: gentamicin treatment for 4 days or 7 days; GM + MK 4d, 7d: combined treatment with gentamicin and MK801 for 4 days or 7 days; IHC: inner hair cell; ISBs: inner spiral bundles. Scale bar = 10 *μ*m.

**Figure 6 fig6:**
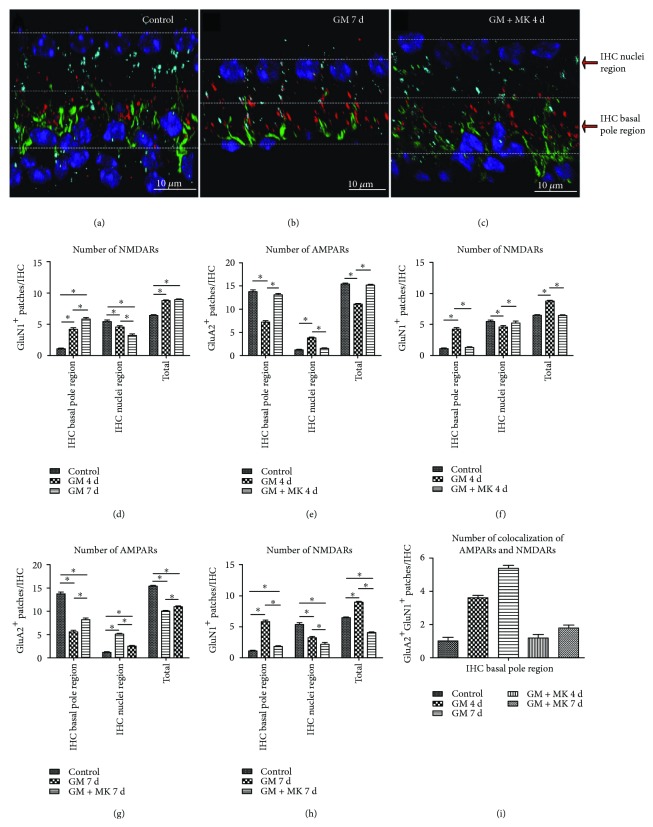
The 3D-reconstructed images and the numbers of NMDARs and AMPARs at the nerve fibers around the IHCs. Postsynaptic AMPARs, NMDARs, and nerve fibers around the IHCs were identified by immunostaining for GluA2 (red), GluN1 (cyan), and neurofilament (NF) (green). Nuclei were labeled with DAPI (blue). (a, b, c) In the study, we define the “IHC basal pole region” and the “IHC nuclei region” in every single IHC. The changed tendency in location of AMPARs and NMDARs was observed at the afferent dendrites around the adjacent IHC in the 3D-reconstructed images. (a) The locations of NMDARs and AMPARs at the afferent dendritic terminals in the normal cochlea. (b) AMPARs and NMDARs essentially switched locations on SGN dendrites after gentamicin treatment for 7 days. (c) The gentamicin-induced translocation of AMPARs and NMDARs was almost completely blocked after coinjection of MK801 and gentamicin for 4 days. (d, f, h) The numbers of GluN1-positive NMDARs. (e, g) The numbers of GluA2-positive AMPARs. (i) The numbers of colocalized AMPARs and NMDARs at the basal poles of the IHCs. GM 4 d, 7 d: gentamicin treatment for 4 days or 7 days; GM + MK 4d, 7d: combined treatment of gentamicin and MK801 for 4 days or 7 days; IHC: inner hair cell. ^∗^ *p* < 0.05.

We next explored the changes in NMDARs and its relationship with AMPARs after gentamicin injury. First, we found that the number of NMDARs was significantly increased in the SGN-IHC synapses after gentamicin treatment (*p* < 0.05) (Figures 5(b), 5(d), 6(b), and 6(d)). Second, we observed the rearrangement of NMDARs at afferent dendritic terminals. NMDAR patches migrated towards the basal poles of the IHCs, closer to the ISBs. After gentamicin treatment, compared with the control group, the number of NMDAR patches significantly increased in the IHC basal pole region (*p* < 0.05) and significantly decreased in the IHC nuclei region (*p* < 0.05) (Figures 5(b), 5(d), 6(b), and 6(d) and 4 days, the numbers of NMDARs were 4.50 ± 0.30 and 4.20 ± 0.31 per IHC in the IHC nuclei region and the IHC basal pole region, respectively (Figures 5(b) and 6(d), and Supplementary Table 2). After gentamicin treatment for 7 days, the numbers of NMDARs were 3.20 ± 0.25 and 5.80 ± 0.25 per IHC in the IHC nuclei region and the IHC basal pole region, respectively (Figures 5(d), 6(b), and 6(d) and Supplementary Table 2). Moreover, the number of colocalized AMPARs and NMDARs per IHC was also significantly increased in the IHC basal pole region after gentamicin treatment (Figure 6(i) and Supplementary Table 2).

### 3.4. Treatment with the NMDAR Antagonist MK801 Prevented the Gentamicin-Induced Rearrangement of AMPARs and NMDARs at the IHC-SGN Synaptic Connection

MK801 is a noncompetitive NMDA receptor antagonist that is thought to protect neurons in the brain against excitotoxicity induced by excessive glutamate activity [17, 32]. MK801 was used as a NMDAR antagonist with the dose range from 0.2 to 1.0 (mg/kg) in previous studies [17, 21, 22].To explore the role of NMDARs in synaptic plasticity during ototoxicity, MK801 (0.2 mg/kg, i.p.) was used to rescue the gentamicin-induced damage. Briefly, mice were injected daily with MK801 and/or gentamicin for 4 or 7 consecutive days, and mice receiving injections of normal saline served as controls. ABR tests were performed on the following day after injections for 4 or 7 days.

GluN1 staining showed that MK801 treatment clearly prevented the gentamicin-induced movement of NMDARs toward the IHC basal poles (Figures 5 and 6). The number, morphology, and distribution of GluN1-positive patches in MK801-treated cochleae were similar to normal cochleae (Figure 6). Compared with the gentamicin-only group, the number of NMDARs in the IHC nuclei region increased significantly in the MK801 rescue group in both the 4-day and 7-day treatment groups (Figures 6(f) and 6(h)). There was no significant difference in the number of NMDARs in the IHC nuclei region between the undamaged control group and the MK801 rescue group in the 4-day treatment group (Figure 6(f)).

**Figure 7 fig7:**
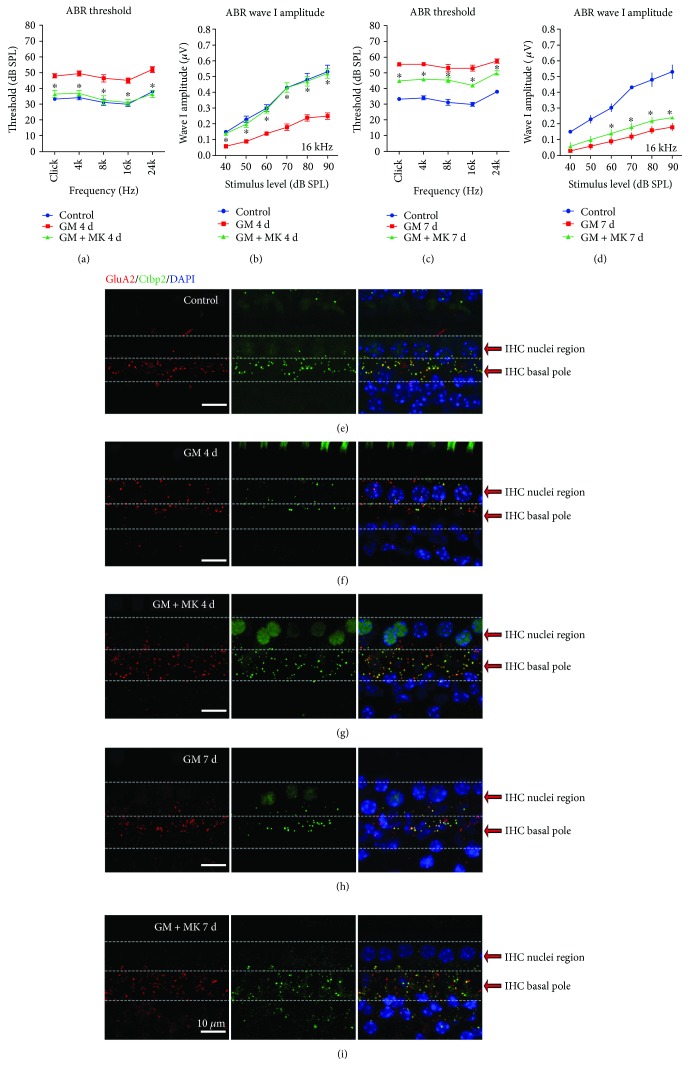
Cochlear ribbon synapses were maintained when MK801 was coinjected with gentamicin. (a-b) The elevation of ABR thresholds and the decline of ABR wave I amplitudes induced by gentamicin injury were successfully rescued by MK801 treatment in the 4-day treatment group (^∗^ *p* < 0.05, *versus* gentamicin-only group). (c-d) The elevation of ABR thresholds and the decline of ABR wave I amplitudes induced by gentamicin injury were partly blocked by MK801 treatment in the 7-day treatment group (^∗^ *p* < 0.05, *versus* gentamicin-only group). (e–i) Afferent ribbon synaptic pairs were observed by immunostaining presynaptic ribbons with anti-CtBP2 (green) and postsynaptic AMPARs with anti-GluA2 (red). Nuclei were labeled by DAPI (blue). (e) Ribbons and AMPARs were paired at the basal poles of the IHCs in the undamaged cochlea. (f) After gentamicin treatment for 4 days, AMPARs and ribbons moved upwards towards the bundle poles of the IHCs. The translocation of AMPARs occurred earlier than that of the presynaptic ribbons. (g) After the combined treatment with gentamicin and MK801 for 4 days, the ribbon synaptic pairs had similar location, morphology, and distribution as undamaged controls. (h) After gentamicin treatment for 7 days, AMPARs and ribbons were further relocated towards the bundle poles of the IHCs. Some large ribbons and/or AMPARs were also observed at the basal poles of the IHCs. (i) After the combined treatment with gentamicin and MK801 for 7 days, the quantity of the ribbon synaptic pairs was significantly increased in the IHC basal pole region compared with that in gentamicin-only treatment. GM 4 d, 7 d: gentamicin treatment for 4 days or 7 days; GM + MK 4d, 7d: combined treatment with gentamicin and MK801 for 4 days or 7 days; IHC: inner hair cell. Scale bar = 10 *μ*m. ^∗^ *p* < 0.05.

**Figure 8:**
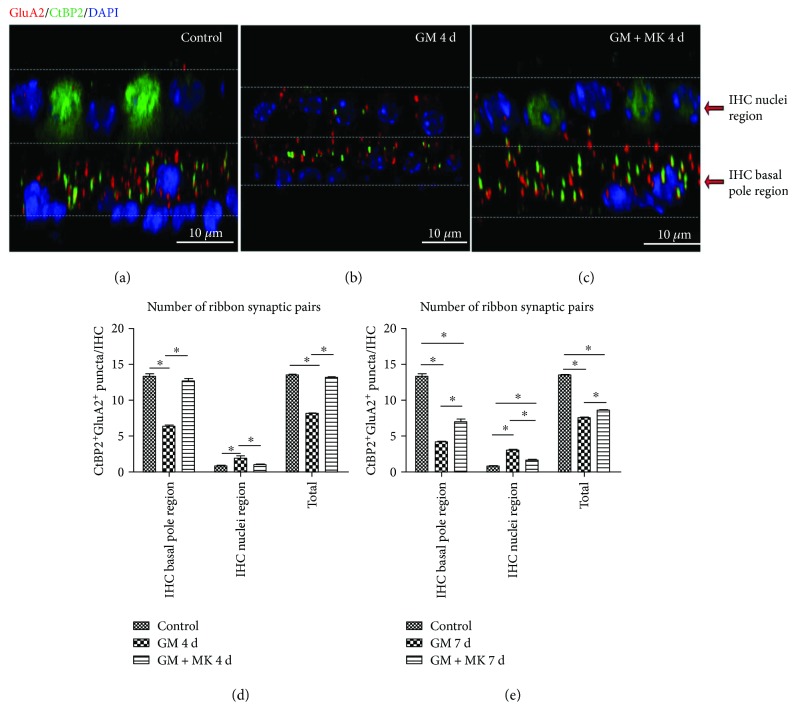
The 3D-reconstructed images and numerical data for the ribbon pairs after different treatments. Afferent synapses on IHCs are seen by immunostaining presynaptic ribbons with anti-CtBP2 (green) and postsynaptic AMPARs with anti-GluA2 (red). Nuclei were labeled with DAPI (blue). (a, b, c) In the study, we define the “IHC basal pole region” and the “IHC nuclei region” in every single IHC. The changed tendency in location of AMPARs and ribbons was observed around the IHC in the 3D-reconstructed images. (a) Ribbons and AMPARs were paired at the basal poles of the IHCs in the control cochlea. (b) After gentamicin treatment for 4 days, the presynaptic ribbons and postsynaptic AMPARs were relocated towards the bundle poles of the IHCs. The number of ribbons and AMPARs was decreased at the basal pole of IHCs. (c) After combined treatment with gentamicin and MK801 for 4 days, most of the IHC ribbons and postsynaptic AMPARs remained at the basal poles of the IHCs, but a few AMPARs were still observed near the IHC nuclei region. (d) The number of ribbon synaptic pairs among the control group, gentamicin treatment group for 4 days, and combined treatment group with gentamicin and MK801 for 4 days. (e) The number of ribbon synaptic pairs among the control group, gentamicin treatment group for 7 days, and combined treatment group with gentamicin and MK801 for 7 days. GM 4 d, 7 d: gentamicin treatment for 4 days or 7 days; GM + MK 4d, 7d: combined treatment with gentamicin and MK801 for 4 days or 7 days; IHC: inner hair cell ^∗^ *p* < 0.05.

GluA2 staining showed that the gentamicin-induced movement toward the IHC bundle poles and the pathological changes of postsynaptic AMPARs were blocked by coinjection of MK801 (Figures 5 [Fig fig6] [Fig fig7]–8). Moreover, compared with the gentamicin-only group, the number of colocalized AMPARs and NMDARs significantly decreased in the IHC basal pole region in the MK801 rescue group in both the 4-day and 7-day treatment groups (*p* < 0.05, Figures 6(f), 6(h), and 6(i)). These results suggested that MK801 treatment blocked the gentamicin-induced activation of NMDARs and the pathological changes of AMPARs in the SGN terminals.

### 3.5. NMDAR Antagonist MK801 Treatment Protects against Gentamicin-Induced Hearing Loss by Preventing the Disruption of Ribbon Synapses

We next explored whether gentamicin-induced hearing loss and disruption of ribbon synaptic pairs could be affected by the NMDAR antagonist MK801. In this experiment, we found that gentamicin-induced hearing loss was successfully rescued by MK801 treatment (Figures 7(a)–7(d)). Compared with the undamaged group, the ABR thresholds were significantly increased while ABR wave I amplitudes were significantly reduced after gentamicin injury. However, the elevation of ABR thresholds and reduction of wave I amplitudes induced by gentamicin injury was successfully rescued by MK801 treatment in both the 4-day and 7-day treatment groups. Indeed, no significant differences were seen in the ABR thresholds and wave I amplitude between the undamaged control group and the MK801 rescue group in the 4-day treatment group (Figures 7(a) and 7(b)), suggesting that MK801 treatment could attenuate the gentamicin-induced hearing loss.

CtBP2 staining showed that the movement of presynaptic ribbons toward the IHC bundle poles in response to gentamicin was blocked by coinjection with MK801 (Figures 7 and 8). Not only the location but also the quantity, morphology, and distribution of presynaptic ribbons in the MK801-treated cochlea were comparable to normal undamaged cochleae (Figures 7(e)–7(i), and Supplemental Video 5). Compared with the gentamicin-only group, the numbers of ribbon synaptic pairs at the IHC basal pole region were significantly increased in the MK801 rescue groups at both 4 and 7 days (Figures 8(d) and 8(e)). There was no significant difference in the number of ribbon synaptic pairs in the IHC basal pole region between the control and the MK801 rescue group in the 4-day treatment group (Figure 8(d)). These results suggested that the rearrangement of NMDARs is a primary element of the injury in IHC-SGNs synapses induced by gentamicin and early interruption of NMDAR activation protected against gentamicin-induced pathological changes in the IHC synapses. Together, these results suggest that inhibition of NMDARs protected against gentamicin-induced hearing loss, likely by maintaining the integrity of the ribbon synapses.

## 4. Discussion

Transmission of nerve impulses at IHC-SGN synapses is mediated by the release of glutamate from ribbon terminals [2, 33]. Patch clamp recordings of the type I SGN afferent dendrite convincingly show that excitatory postsynaptic currents are AMPAR mediated and that NMDARs do not contribute to synaptic transmission at the type I SGN synapse under physiological conditions [34–37]. Although the role of NMDARs in ototoxicity was speculated to be through glutamate excitotoxicity [15–17], there was no direct morphological evidence for this or for the role of NMDARs in cochlear IHC-SGN synapses.

### 4.1. NMDARs and AMPARs Are Located in Different Regions of ANF Terminals under Physiological Conditions

GluA2, GluA3, and possibly GluA4 subunits of AMPAR are present in SGN afferent dendrites in the adult cochlea [38], and the GluA2 subunit determines the key biophysical properties of GluAs *in vivo* [39]. Under physiological conditions, GluA2-positive AMPAR patches were paired with CtBP2 positive IHC ribbons and were distributed in the ISBs around the basal poles of the IHCs (Figure 4), which was consistent with previous studies [2, 3, 8]. NMDARs are composed of the mandatory GluN1 subunit and a variety of GluN2A, B, C, and D subunits [38, 40]. In this paper, we found that the majority of NMDARs were distributed on the modiolar side and close to the nuclei region of IHCs. However, the AMPARs are mainly distributed at the IHC basal pole region, on the modiolar and pillar sides of IHCs. Some NMDARs were colocalized with small GluA2-positive puncta, which might be regarded as a “resting state” of small-puncta AMPARs in the central nervous system (Figure 1) [38, 41]. This study reported the different characteristics of the distribution of AMPARs and NMDARs at type I ANFs contacting IHCs in the adult mouse cochlea (Figures 1 and 5).

### 4.2. NMDARs Are Involved in AMPAR Rearrangement in the Cochlear IHC-SGN Synapse Connection as Part of the Ototoxic Mechanism of Gentamicin Treatment

**Figure 9:**
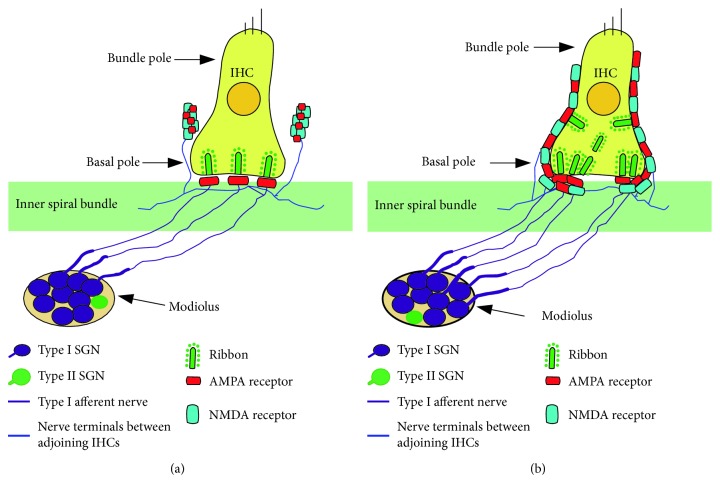
Diagrams illustrating the gentamicin-induced changes in ribbons, NMDARs, and AMPARs at the IHC-SGN synapse in the mouse cochlea. (a) In the normal mouse adult cochlea, presynaptic ribbons and postsynaptic AMPARs show a nearly one-to-one relationship. The ribbon synaptic pairs (double staining for CtBP2-positive puncta and GluA2-positive patches) are distributed around the basal poles of the IHCs. Most of the AMPARs are observed in the ISBs, but most NMDARs are distributed on the neural dendritic terminals between the adjoining IHCs and closer to the IHC nuclei region. In IHC nuclei region, some NMDARs colocalize with GluA2-positive AMPAR puncta, which are smaller than AMPAR patches in the IHC basal pole region. In the mouse cochlea, each IHC is contacted by roughly 10–20 ANFs depending on cochlear location. Each cochlear neuron is excited by a single ribbon synapse with a single IHC. (b) After gentamicin treatment, AMPARs and NMDARs are relocated at nerve fiber terminals around IHCs, and their locations are essentially reversed. AMPARs move upwards towards the bundle poles of the IHCs, and NMDARs migrate downwards towards the basal poles of the IHCs. The number of colocalized AMPARs and NMDARs gradually increases in the IHC basal pole region. Some anomalously aggregated ribbons and/or AMPARs in the IHC basal pole region are observed. IHC: inner hair cell; SGN: spiral ganglion neuron; ISBs: inner spiral bundles; ANFs: afferent nerve fibers.

Reorganization of postsynaptic AMPARs and NMDARs was observed after gentamicin treatment. Rows of NMDARs moved towards the IHC basal poles, while AMPAR patches moved towards the IHC bundle poles and reached to or across the IHC nuclei region on the afferent dendrites contacting the IHCs (Figures 5, 6, and 9). As a result, the spatial distribution AMPARs and NMDARs was different from the distribution under physiological conditions. The number of NMDARs in the ISBs was significantly increased in response to gentamicin treatment (Figures 5 and 6). In the central nervous system, calcium overload and cell death are mediated by NMDARs through glutamate excitotoxicity [40, 42–44], and our data suggest that rearrangement of AMPARs and NMDARs might be involved in the glutamate excitotoxicity observed in cochlear IHC-SGN synapses after gentamicin treatment.

In the brain, NMDARs are required for the control of synaptic rearrangement and axonal remodeling [45]. Activation of synaptic NMDARs induces the membrane insertion of new AMPARs in cultured hippocampal neurons [46], and NMDAR agonists can regulate the expression of surface AMPARs on the cell membrane of cultured SGNs [14]. In the present study, coinjection of the NMDAR antagonist MK801 and gentamicin prevented the rearrangement of AMPARs and NMDARs at the dendritic terminals (Figures 5 [Fig fig6] [Fig fig7]–8), suggesting that NMDAR activation is involved in the AMPAR rearrangement in IHC-SGN synapses in response to gentamicin. Furthermore, cotreatment with MK801 prevented gentamicin-induced damage of the IHC ribbon synapse (Figure 7) and reduced gentamicin-induced hearing loss (Figure 8), suggesting that NMDARs are involved in ototoxicity by regulating the number and distribution of ribbon synapses in the IHC-SGN afferent synapse connection.

### 4.3. Ribbons in IHCs Might Follow the Rearrangement of AMPARs at Afferent Dendritic Terminals in Response to Gentamicin Treatment

Studies of both noise-induced hearing loss and drug-induced hearing loss have shown reduced numbers and abnormal distributions of ribbons, which were found to be isolated in the cytoplasm and proximal to the IHC nuclei region [7, 8]. However, the mechanism and implications of this abnormal distribution of ribbons has remained unclear.

Under physiological conditions, presynaptic ribbons were paired to postsynaptic AMPARs. In gentamicin-induced ototoxicity, AMPARs on the SGN dendrites moved quickly toward the bundle poles of the IHCs, some of which reached to or across the IHC nuclei region. Orphan AMPARs lacking opposed ribbons were often observed in the IHC nuclei region of SGN afferent dendritic terminals in the 4-day gentamicin group. However, in the 7-day gentamicin group, the IHC ribbons were increasingly relocated and matched up again with postsynaptic AMPARs in the IHC nuclei region. These results suggest that the relocation of AMPARs takes place earlier than the relocation of the presynaptic ribbons and that the presynaptic ribbons in the IHC membrane might follow the movement of postsynaptic AMPARs at type I SGN afferent dendritic terminals in response to gentamicin treatment (Figure 4).

The expression of mature ribbons on the membrane of IHCs requires several intracellular processes, including the synthesis of glutamate vesicles, transportation, assembly, and final localization of ribbons at the membrane [2]. Sobkowicz et al. studied the distribution of synaptic ribbons in the developing organ of Corti and found that nerve fibers appear to be critical in influencing the location of the synaptic ribbon [47]. It has been suggested that nitric oxide, as a neuronal messenger, might be involved in transferring signals between presynaptic and postsynaptic elements and regulating the excitability at glutamatergic synapses. In the brain, neuronal nitric oxide synthase is broadly expressed and is associated with synaptic plasticity through NMDAR-mediated calcium influx [48]. The role of nitric oxide and synaptic plasticity in the progression of ototoxicity requires further study.

## 5. Conclusions

This study showed that NMDARs are involved in cochlear ribbon synaptic rearrangement in gentamicin-induced ototoxicity. The postsynaptic arrangement of AMPARs on the dendrites of SGNs might affect the number and location of presynaptic ribbons. Inhibition of NMDARs successfully prevented gentamicin-induced ototoxicity by preventing the relocation of AMPARs and NMDARs on the dendrites of the SGNs and thus maintaining the integrity of the ribbon synapses and preserving hearing function.

## Data Availability

The data used to support the findings of this study are available from the corresponding author upon request.

## Supplementary Materials

Supplementary Materials Supplementary Table 1: the number of presynaptic ribbons, postsynaptic AMPARs, and ribbon synaptic pairs in different groups. Supplementary Table 2: the number of the AMPARs and NMDARs at the IHC-SGN synapse in different groups. Legends of supplemental videos: Supplemental Video 1: spatial distribution of NMDARs and AMPARs in the IHC-SGN synapse of normal mouse mature cochlea. Supplemental Video 2: spatial distribution of NMDARs and AMPARs in the IHC-SGN synapse of gentamicin treated mouse cochlea (GM 7d). Supplemental Video 3: spatial distribution of ribbons and AMPARs in the IHC-SGN synapse of normal mouse mature cochlea. Supplemental Video 4: spatial distribution of ribbons and AMPARs in the IHC-SGN synapse of gentamicin-treated mouse cochlea (GM 4d). Supplemental Video 5: spatial distribution of ribbons and AMPARs in the IHC-SGN synapse of gentamicin and MK801-treated mouse cochlea (GM + MK 4d).
