# Supplementary material for: N-Methyl-D-Aspartate Receptors Involvement in the Gentamicin-Induced Hearing Loss and Pathological Changes of Ribbon Synapse in the Mouse Cochlear Inner Hair Cells
Source: Neural Plast. 2018 Jul 15;2018:3989201. doi: 10.1155/2018/3989201 (PMC6079453; doi:10.1155/2018/3989201)
Supplement: Supplementary Materials — Supplementary Table 1: the number of presynaptic ribbons, postsynaptic AMPARs, and ribbon synaptic pairs in different groups. Supplementary Table 2: the number of the AMPARs and NMDARs at the IHC-SGN synapse in different groups. Legends of supplemental videos: Supplemental Video 1: spatial distribution of NMDARs and AMPARs in the IHC-SGN synapse of normal mouse mature cochlea. Supplemental Video 2: spatial distribution of NMDARs and AMPARs in the IHC-SGN synapse of gentamicin treated mouse cochlea (GM 7d). Supplemental Video 3: spatial distribution of ribbons and AMPARs in the IHC-SGN synapse of normal mouse mature cochlea. Supplemental Video 4: spatial distribution of ribbons and AMPARs in the IHC-SGN synapse of gentamicin-treated mouse cochlea (GM 4d). Supplemental Video 5: spatial distribution of ribbons and AMPARs in the IHC-SGN synapse of gentamicin and MK801-treated mouse cochlea (GM + MK 4d). [file 3989201.f1.zip › supplemental materiels.pdf]

Supplemental Table 1 The number of presynaptic ribbons, postsynaptic AMPARs, and ribbon synaptic pairs in different groups. Data were shown as means ±SE.

Groups (n=5)	Number of ribbons (per IHC)			Number of AMPARs (per IHC)			Number of ribbon synaptic pairs (per IHC)		
	Basal pole	Nuclei region	Total	Basal pole	Nuclei region	Total	Basal pole	Nuclei region	Total
control	13.30±0.37	0.80±0.10	14.12±0.20	13.70±0.42	1.20±0.13	15.30±0.24	13.30±0.37	0.80±0.10	13.50±0.12
GM4d	6.40±0.37	2.20±0.32	8.90±0.24	7.20±0.39	3.80±0.28	11.00±0.21	6.30±0.31	1.90±0.32	8.10±0.24
GM7d	4.20±0.23	3.10±0.28	7.50±0.21	5.50±0.37	5.00±0.31	10.02±0.23	4.20±0.23	3.00±0.28	7.50±0.21
GM+MK 4d	12.70±0.40	1.00±0.13	13.80±0.30	13.00±0.39	1.50±0.17	15.02±0.30	12.70±0.30	1.00±0.13	13.20±0.20
GM+MK 7d	7.00±0.32	1.60±0.20	8.80±0.24	8.20±0.37	2.50±015	11.00±0.20	7.00±0.32	1.60±0.20	8.60±0.24

GM 4d, 7d: gentamicin treatment for 4 days or 7 days; GM+MK 4d, 7d: combined treatment with gentamicin and MK801 for 4 days or 7 days; IHC: inner hair cell.

Supplemental Table 2 The number of the AMPARs and NMDARs at the IHC-SGN synapse in different groups. Data were shown as means ±SE.

Groups (n=5)	Number of AMPARs (per IHC)			Number of NMDARs (per IHC)			Number of the co-localization of the AMPAR and NMDAR (per IHC)
	Basal pole	Nuclei region	Total	Basal pole	Nuclei region	Total	Basal pole
control	13.70±0.42	1.20±0.13	15.30±0.24	1.00±0.22	5.40±0.27	6.40±0.21	1.00±0.12
GM4d	7.20±0.39	3.80±0.28	11.00±0.21	4.20±0.31	4.50±0.30	8.70±0.20	3.60±0.16
GM7d	5.50±0.37	5.00±0.31	10.02±0.23	5.80±0.25	3.20±0.25	8.90±0.18	5.38±0.17
GM+MK 4d	13.00±0.39	1.50±0.17	15.02±0.30	1.20±0.21	5.20±0.34	6.34±0.20	1.20±0.21
GM+MK 7d	8.20±0.37	2.50±015	11.00±0.20	1.80±0.17	2.20±0.27	4.00±0.17	1.80±0.17

GM 4d, 7d: gentamicin treatment for 4 days or 7 days; GM+MK 4d, 7d: combined treatment with gentamicin and MK801 for 4 days or 7 days; IHC: inner hair cell.

Legends of supplemental videos:

Supplemental video 1: Spatial distribution of NMDARs, AMPARs in the IHC-SGN synapse of normal mouse mature cochlea. Postsynaptic AMPARs, NMDARs, and nerve fibers were identified by immunostaining for GluA2 (red), GluN1 (cyan), and NF (green), respectively. Nuclei were labeled with DAPI (blue).

Supplemental video 2: Spatial distribution of NMDARs, AMPARs in the IHC-SGN synapse of gentamicin treated mouse cochlea (GM 7d). Postsynaptic AMPARs,

NMDARs, and nerve fibers were identified by immunostaining for GluA2 (red), GluN1 (cyan), and NF (green), respectively. Nuclei were labeled with DAPI (blue).

Supplemental video 3: Spatial distribution of ribbons and AMPARs in the IHC-SGN synapse of normal mouse mature cochlea. Presynaptic ribbons and postsynaptic AMPARs were identified by immunostaining for CtBP2 (green) and GluA2 (red), respectively. Nuclei were labeled with DAPI (blue). The cell nuclei in the upper row belonged to inner hair cells.

Supplemental video 4: Spatial distribution of ribbons and AMPARs in the IHC-SGN synapse of gentamicin treated mouse cochlea (GM 4d). Presynaptic ribbons and postsynaptic AMPARs were identified by immunostaining for CtBP2 (green) and GluA2 (red), respectively. Nuclei were labeled with DAPI (blue). The cell nuclei in the upper row belonged to inner hair cells.

Supplemental video 5: Spatial distribution of ribbons and AMPARs in the IHC-SGN synapse of gentamicin and MK801 treated mouse cochlea (GM+MK 4d). Presynaptic ribbons and postsynaptic AMPARs were identified by immunostaining for CtBP2 (green) and GluA2 (red), respectively. Nuclei were labeled with DAPI (blue). The cell nuclei in the lower row belonged to inner hair cells.
